# Finite Element Analysis of the Cingulata Jaw: An Ecomorphological Approach to Armadillo’s Diets

**DOI:** 10.1371/journal.pone.0120653

**Published:** 2015-04-28

**Authors:** Sílvia Serrano-Fochs, Soledad De Esteban-Trivigno, Jordi Marcé-Nogué, Josep Fortuny, Richard A. Fariña

**Affiliations:** 1 Institut Català de Paleontologia M. Crusafont, Cerdanyola del Valles, Catalonia, Spain; 2 Universitat Politècnica de Catalunya, Terrassa, Catalonia, Spain; 3 Paleontología, Facultad de Ciencias, Universidad de la República, Montevideo, Uruguay; 4 Transmitting Science, Piera, Spain

## Abstract

Finite element analyses (FEA) were applied to assess the lower jaw biomechanics of cingulate xenarthrans: 14 species of armadillos as well as one Pleistocene pampathere (11 extant taxa and the extinct forms *Vassallia*, *Eutatus* and *Macroeuphractus*). The principal goal of this work is to comparatively assess the biomechanical capabilities of the mandible based on FEA and to relate the obtained stress patterns with diet preferences and variability, in extant and extinct species through an ecomorphology approach. The results of FEA showed that omnivorous species have stronger mandibles than insectivorous species. Moreover, this latter group of species showed high variability, including some similar biomechanical features of the insectivorous *Tolypeutes matacus* and *Chlamyphorus truncatus* to those of omnivorous species, in agreement with reported diets that include items other than insects. It remains unclear the reasons behind the stronger than expected lower jaw of *Dasypus kappleri*. On the other hand, the very strong mandible of the fossil taxon *Vassallia maxima* agrees well with the proposed herbivorous diet. Moreover, *Eutatus seguini* yielded a stress pattern similar to *Vassalia* in the posterior part of the lower jaw, but resembling that of the stoutly built *Macroeuphractus outesi* in the anterior part. The results highlight the need for more detailed studies on the natural history of extant armadillos. FEA proved a powerful tool for biomechanical studies in a comparative framework.

## Introduction

The mammalian superorder Xenarthra comprises a group of peculiar, mostly South American, placental mammals forming a monophyletic group with highly heterogeneous morphology. It includes the orders Cingulata and Pilosa, which showed a huge disparity of forms [1,2], most of them extinct. As a consequence of many Pleistocene xenarthrans having attained very large and even gigantic size, they turned out to be vulnerable in the Quaternary mass extinction about 10,000 years ago [3], and the current taxonomic diversity of this group is much reduced [4–5].

Herein, we will focus on the armored xenarthrans, the Cingulata, which contain three major groups: armadillos (Dasypodidae, a paraphyletic group [6], giant extinct armadillos (Pampatheriidae) and glyptodonts (Glyptodontidae).

Living representatives of cingulates belong to Dasypodidae, and are included in 21 extant species [7] and nine genera [8] within the currently accepted subfamilies: Dasypodinae (*Dasypus*), Euphractinae (*Euphractus*, *Chaetophractus* and *Zaedyus*), Chlamyphorinae (*Calyptophractus* and *Chlamyphorus*) and Tolypeutinae (*Tolypeutes*, *Priodontes*, and *Cabassous*) [8].

In comparison with other mammals, the Xenarthra present peculiar features that define the clade; the most distinctive trait within Cingulata is the presence of carapace, which differs morphologically in the different groups. Other peculiar features are observed in the dental anatomy: simplified teeth, characterized by absence of enamel, homodonty and hypsodonty are present in almost all known species [9]. The dietary habits of extant Cingulata are mainly known from stomach contents (e.g. [10]) although in some species our knowledge is still scarce and limited. Otherwise, the absence of enamel hinders paleontological inferences for diet of extinct forms by using dental microwear analysis, although some attempts have been done in xenarthrans [11–14].

Although it has been proposed that diet in armadillos might be difficult to predict from the shape of the masticatory apparatus, as some species will be able to fed on items for which their morphology is not optimal [15], Vizcaíno and coauthors [16] state that despite mammals can subsist on food items other than those in their preferred diet, the dietary preferences of the different species are in agreement with what is expected from their morphology. Indeed, biomechanical and ecomorphological analyses have been undertaken in order to infer the diet of extinct cingulates from the shape of their jaw and skull [1, 9, 16–23]. However, in most cases the lack of similar extant forms makes ecomorphological inferences difficult.

In this article we propose a new insight to deepen on the knowledge of the relationship between morphology and feeding ecology of Cingulata. To carry out this goal, Finite Element Analysis (FEA) was used to analyze the mandibles of 14 species of Cingulata. FEA is a noninvasive modeling technique that is based on a numerical analysis and the principle of dividing a system into a finite number of discrete elements [24–25] on which the equations are applied. Mechanical properties of these elements are defined in order to give the structure a realistic behavior as well as constrain the model to anchor it in space, including muscular loads. FEA models enable the observation of stress distribution patterns of the specimens (in this case, each representing the mandible of each species) by simulating loadings and forces involved in the masticatory function. Under equivalent loads, these stress patterns can be interpreted as a sign of the relative strength, with specimens (xenarthran mandibles, in our case) with higher stress being weaker. Assuming that more robust or stronger mandibles would be needed both for processing harder food items, insect-feeding armadillos (which have none or little processing on the mouth) should be expected to have weaker mandibles (i.e. with higher stress levels) than those feeding on other items, such as herbivorous or omnivorous species. On the other hand, differences in stress distribution pattern may give a clue on different aspects of the feeding ecology of the analyzed species.

In particular, the aims of this study were: 1) To evaluate the biomechanical behavior of the mandible of some extant and extinct armadillos and one pampatherid under certain loads; 2) to test if there is any relationship between the stress patterns of the lower jaw and the proposed diets in extant and extinct species.

For doing so, planar models of mandibles belonging to several cingulate species were analyzed through FEA methods to understand the adaptive significance of this biological structure [25] and why evolution shaped it in that particular manner [26].

## Materials and Methods

In this study 14 cingulate species have been analyzed, eleven of which are extant (Table 1). The original material consists in lateral images of mandibles of armadillos taken from the specimens housed in different museums by S.D.E.T. (see Table 1). Images from the extinct species were obtained from the literature: *Vassallia maxima* Late Miocene-Early Pliocene, FMNH 14424 [17], although it was first-hand observed by one of the authors (S.D.E.T.). *Macroeuphractus outesi* Late Pliocene (MLP 69-IX-9-3)/ (MLP 64-VIII-25-1; [27–28]. *Eutatus seguini* Late Pliocene to Early Holocene, reconstruction made by Vizcaíno and Bargo [17].

**Table 1:** List of extant extant species used in the present study.

Taxon	Collection number	Subfamily	Redford (1985) category
*Tolypeutes matacus*	AMNH 246460	Tolypeutinae	Generalist insectivore
*Priodontes maximus*	AMNH 208104	Tolypeutinae	Specialist insectivore
*Cabassous unicinctus*	MNHN 1953/457	Tolypeutinae	Specialist insectivore
*Chlamyphorus truncatus*	ZMB 4321	Chlamyphorinae	Generalist insectivore
*Dasypus sabanicola*	ZMB 85899	Dasypodinae	Generalist insectivore
*Dasypus kappleri*	MNHN 1995/207	Dasypodinae	Generalist insectivore
*Dasypus novemcinctus*	AMNH 133338	Dasypodinae	Generalist insectivore
*Chaetophractus vellerosus*	MLP 18.XI.99,9	Euphractinae	Omnivore/ Carnivore
*Chaetophractus villosus*	MNCN 2538	Euphractinae	Omnivore/ Carnivore
*Euphractus sexcinctus*	MNHN 1917/13	Euphractinae	Omnivore/ Carnivore
*Zaedyus pichiy*	MLP 9.XII.2.10	Euphractinae	Omnivore/ Carnivore

Diet following Redford (1985): Specialist insectivores (social insects, mainly ants and termites), Generalist insectivores (beetles, beetle larvae, spiders, ants, termites), Omnivores/ Carnivores (characterized by a diet including plant material-e.g. tubers, roots, palm, nuts-, animal matter and a variety of invertebrates-beetles, ants, termites, worms- and vertebrates-mice, carrion, birds, eggs-). Abbreviations preceding the names of institutions are used to identify the location of specimens. AMNH, American Museum of Natural History, New York, USA; FMNH, Field Museum of Natural History, Chicago, USA; MNCN, Museo Nacional de Ciencias Naturales, Madrid, Spain; MNHN, Muséum National d’Histoire Naturalle, Paris, France; ZMB, Zoologisches Museum, Berlin, Germany; MLP, Museo de la Plata, La Plata, Argentina.

### Diet

The knowledge of the diet allows placing the species within an ecological context. Herein, we classified each species according to a range of trophic specialization into one of the three diet groups most widely accepted in armadillo literature: Specialized insectivorous, generalist insectivorous and omnivorous/carnivorous. That classification of each species was made on the basis of the current knowledge in the ecology of armadillos, mainly based on stomachs contents [10,29–37] (Table 1).

The extinct taxa analysed here have been subject of phylogenetic and morphological studies [16–17]. From a paleobiological viewpoint, the pampatheriid *V*. *maxima* has been proposed as an herbivorous species, primarily grazer, consuming mainly coarse vegetation [9, 17, 28]. It should be noted that herbivores were not included in the categories of extant xenarthrans, because there is no extant species showing this behavior. Additionally, different diets have been proposed for the two extinct dasypodids (*E*. *seguini* and *M*. *outesi*). On one hand, *E*. *seguini* has been also proposed as an herbivore [16], whilst *Macroeuphractus outesi* has been considered like the most carnivorous extreme in the range of carnivore-omnivore [18, 28].

### Finite Element Analysis

Plane models of the mandible of 14 species of armadillos were analyzed, and stress patterns were obtained using FEA. In continuum mechanics, plane elasticity refers to the study of particular solutions of the general elastic problem in bodies that are geometrically mechanical prisms (that is, an area with a constant thickness) [38]. In particular, a plane stress solution occurs in structural elements where one dimension (the thickness) is very small compared to the other two, and the stresses are negligible with respect to the smaller dimension. Planar models are commonly used in paleontology and biology [39–41] and here have been applied to the construction of the 14 FEA models. The thickness of each mandible was based in the mean value of three measurements in different points of the actual jaws.

Our goal was generate a model as simple as possible that better predicts and explains the relationships between the variables under study. There is always a trade-off between the reality and the predictive power, as more close to reality implies more complex model (includes more variables) and cannot be used to generalize or to predict the studied relationship on different taxa. In sum, a complex model is very useful for a descriptive purpose, or to check the exact behavior of a biological structure under different situations, but when the final goal is to extract a general relationship and/or predict the ecological patterns on different taxa in a comparative framework (i.e. including many different species in the study), as the present case, simpler models are preferred. The discrimination between different diets in armadillos with such simple models is actually clearer rather than in complex models.

The steps for model construction were modified from the methodology summarized by Fortuny *et al*. [42, 43]: 1) Each photograph or reconstruction was treated with Photoshop v.8.0.1 software to obtain the mandible profile and match the mandible with another picture of the cranium in lateral view, in order to place the masseter and temporalis muscles in both bone elements to obtain later the vector directions of each muscle. 2) Each image was digitized, scaled and outlined using XY coordinates with Image J v.1.36b software, developed at the U.S. National Institute of Health (NIH); 3) Rhinoceros v.4.0 software was used to create a planar surface of each profile; 4) the surface was improved and smoothed using SolidWorks 2012 and imported into Rhinoceros to place and fix muscles and other needed points (see below) on the solid model; and 5) both the creation of the FEA models as well as the recording of the stress distribution results for each mandible were performed. For the generation of the FE mesh, [26], 6-node triangular plane elements (TRI6) and 8-node quadrilateral plane elements (QUAD8) were used, developed with the FEA package Ansys v.12.1 for Windows 7 (64-bit system).

Two main muscles (temporalis and masseter) were considered in the geometry. The insertion area of each muscle was identified and included in the model as a surface. As the amount of force that a muscle can produce depends on its active section [44], the size of the insertion area of each one of the pair of muscles was considered as a proxy for the force each muscle can exert. However, as the interest of the study is a comparative one, instead of calculating absolute values for forces, we used these areas to quantify the proportional amount of force developed by each muscle (see below). In order to compute the direction of the forces, the vectors between the centroids of the corresponding insertion areas when both structures are arranged in life position, were calculated (see below).

For the generation of the FE mesh, [26], triangular and quadrilateral plane elements were created with high level of accuracy and with enough mesh density [45] to capture the variations in the stress patterns and assure the stability of the results. Number of nodes and elements of each cingulate jaw model are in S1 Table.

### Model properties

For the mandibles studied, isotropic and linear elastic bone properties have been assumed. In the absence of data for Cingulata or any close relative, as well as any mammal clade with similar bone structure, the mandible properties of *Macaca mulatta* were used herein; *E* (young’s modulus)-21,000 MPa, *n* (Poissons ratio): 0.45; [46]. The choice of this taxon is because its ecological history; a wide range of habitats and diet comprising plants but also including termites, grasshoppers and ants [47], which resembles thus the omnivorous or generalist insectivorous armadillos.

The thickness of the model has been assumed constant in the whole mandible and obtained from the individual average of three measurements; i) mandibular width at the first jugal, ii) mandibular width at half of the level of antero-posterior length of the jugal series and iii) mandibular width at the posterior end of the jugal series. For the extinct *Macroeuphractus outesi* and *Vassallia maxima*, due to the lack of published measures of thickness, data from *Pampatherium*, the closest taxon with similar cranial-mandible features [17] has been used.

To analyse the stress state results, Von Mises equivalent stresses were obtained for each mandible. The Von Mises criterion is an isotropic criterion used to predict the yielding of ductile materials determining an equivalent state of stress. Considering bone a ductile material [48] and according to [49], when isotropic material properties are defined in cortical bone, the Von Mises criterion is the most adequate for comparing equivalent stress states.

In order to define the location of the landmarks where Von Mises stresses would be recorded, previous simulations were made in three different armadillo’s mandibles, with distinctive features and diets, with the aim to evaluate the distribution and variability of stress patterns. This test enabled us to evaluate the parts of the mandible where there was more variability in the stress distribution and were would be more interesting to record those data.

### Scaling and Masticatory musculature

In order to scale the mandibles of all species to render the forces applied comparable, the quasi-homothetic transformation proposed by Marcé-Nogué *et al*., [50] iwas applied to the FEA models. Eq 1 controls the differences in size holding an equivalent stress distribution between models of 2D Cingulata mandibles in plane elasticity allowing comparisons of the different models avoiding the size and thickness effects.

$$F_{B}=\left(\sqrt{\frac{S_{B}}{S_{B}}}\right)\left(\frac{t_{B}}{t_{A}}\right)F_{A}\;,$$ (Equation 1)

where S_A_ is the area of a reference model, S_B_ the area of a scaled model, T_A_ is the thickness of a reference model and T_B_ the thickness of a scaled model [50].

The musculature used to determine the vector forces in the FEA models includes temporalis and masseter. Muscle reconstruction allowed obtaining the vector direction of the muscular force that was applied to the models (Table 2). The value of the Muscle force was calculated using the Eq 1. An arbitrary force of 1 N was applied to the referenced model.

**Table 2:** Data for those Cingulate species used in the present study regarding the area of the lower jaw, insertion places, forces (musculature force per unit area in N m^-2^), and the thickness and scale factor variables used in the elasticity equation.

Species	Thickness (mm)	Model area [mm^2^]	Masseter area [mm^2^]	Temporalis area [mm^2^]	Masseter Force [N]	Temporalis Force [N]
*Chaetophractus villosus*	4.94	1038.9	300.6	156.1	0.66	0.34
*Priodontes maximus*	6.41	2051.7	616.0	255.1	1.29	0.53
*Cabassous unicinctus*	3.51	415.8	112.1	22.9	0.37	0.08
*Chlamyphorus truncatus*	2.00	113.2	16.0	34.0	0.04	0.09
*Chaetophractus vellerosus*	3.68	538.8	145.0	117.0	0.3	0.24
*Dasypus kappleri*	3.51	971.4	105.4	153.2	0.28	0.41
*Dasypus novemcinctus*	2.94	613.5	225.8	92.2	0.32	0.13
*Dasypus sabanicola*	2.78	527.9	150.7	71.5	0.27	0.13
*Euphractus sexcinctus*	5.66	1019.2	331.2	190.6	0.72	0.41
*Tolypeutes matacus*	3.56	497.4	157.0	64.1	0.35	0.14
*Zaedyus pichiy*	3.51	327.4	89.7	66.1	0.23	0.17
*Vassallia maxima*	25.97	4620.3	2208.4	140.2	10.43	0.66
*Eutatus seguini*	13.74	3517.9	890.5	551.2	3.16	1.96
*Macroeuphractus outesi*	25.97	11750.0	5328.0	1455.9	13.89	3.79

Muscles were modeled from the insertion areas, and the direction of the vector joining the centroid of the muscular attachment in the lower jaw and the centroid of the equivalent muscle attachment in the skull was used as an estimator of force direction. As the objective of this study was to develop a comparative analysis [39], we were not interested in the real value of the forces. On the contrary, our objective was to analyze, under equivalent loads, which mandible show lower stress levels. The value of the muscle force for each model was calculated using the Eq 1 where an arbitrary force of 1 N was applied to *Chaetophractus villosus*, which was the reference model for this study.

On the other hand, in other mammals the proportional development of masseter or temporalis might be indicator of different abilities or diets [51–52]. Therefore, the value of the total force was distributed between the masseter and the temporalis in function of the insertion area (Table 2).

The areas of origin and insertion of the masticatory musculature of the extant armadillos and extinct *Eutatus seguini* were reconstructed from features of the skeleton elements, following Vizcaíno *et al*. [17] and Smith and Redford [15]. Published reconstructions for the main masticatory muscles were used for the extinct *Macroeuphractus outesi* and *Vassallia* [9, 28]

### Boundary Conditions

The boundary conditions were defined and placed, representing the loads, displacements, and constraining anchors that the structure experiences during the some functions [26]. According to the Principle of Saint-Venant, which states that the stresses on a boundary reasonably distant from an applied load are not significantly altered if this load is changed to a statically equivalent load, possible changes in the constraints proposed will have no effect on the results at the landmarks studied.

**Fig 1:**
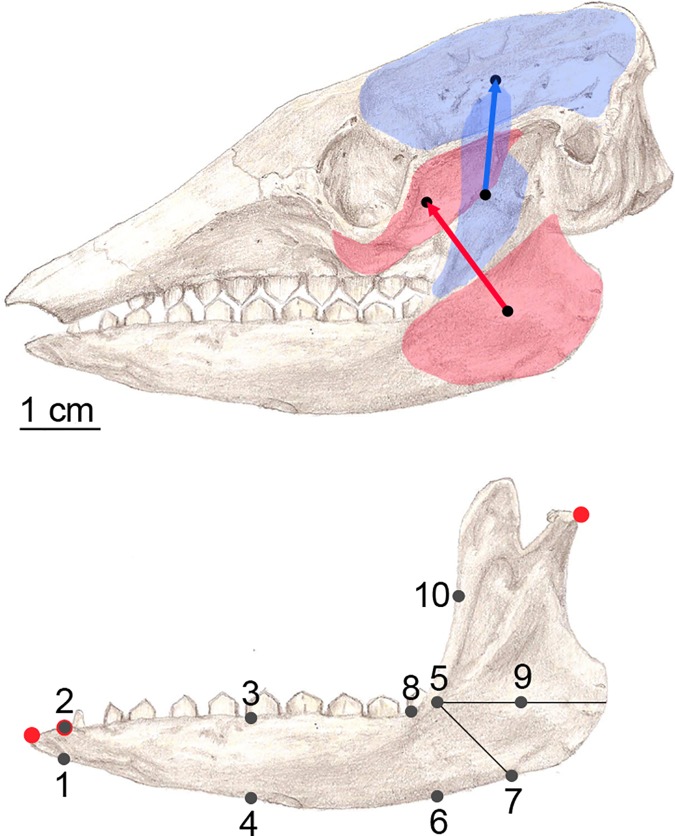
Points where stress values were recorded for posterior analysis. The mandible was oriented making horizontal the line linking the anterior and most posterior part of the tooth row at alveolar level. Landmarks: 1) ventral part of the jaw at the most anterior part of the tooth row, 2) most anterior part of the tooth row at alveolar level, 3) the middle of the tooth row located at alveolar level, 4) drawing a vertical line from the point 3, the point where this line intersects with the ventral part of the mandible, 5) most posterior point of the tooth row at alveolar level, 6) drawing a vertical line from the point 5, the point where this line intersects with the ventral part of the mandible, 7) tracing a line at an angle at 45° from the horizontal, the point where this line intersects with the outer part of the mandibular angle, 8) situated at the upper part of the mandible corpus, where there is an upward shift due to the ascending ramus, 9) tracing an horizontal line that goes trough the point five, the middle point between 5 and the external part of the mandibular ramus, and 10) located between the landmark 5 and the upper part of the coronoid process. The muscle forces were applied in the masseter and the temporalis in directions appropriate for the relative direction of force during chewing. Drawing of *Chaetophractus villosus* mandible.

On the other hand, the values of stress were recorded at specific locations previously defined (landmarks) and later used for quantitative analysis. These data were used in statistical analysis to test for differences between the different diets. These landmarks were defined as biologically homologous as possible between the models (Fig 1).

Two sets were defined simulating different situations; Set 1) simulate the mandible constrained at the most anterior part, Set 2) simulate the mandible constrained at the beginning of the tooth row (Fig 1). The two sets share a constraint on the condyle at the level of the mandibular notch representing the immobilization of the mandible.

### Statistical analysis

Statistical analyses were performed using Past 2.13 [53].

Kruskal-Wallis tests were developed to analyze the relationship between diet and biomechanical properties. This test was applied to each set and landmark, using as groups the classification made at Table 1 for the extant taxa. Extinct taxa were not included in this analysis. Box plots were used for visualizing the results at each landmark.

Two Principal Component Analysis (PCA) on the stress values for all landmarks recorded, developed using the variance-covariance matrix, were carried out for each set. The aim of these analyses was to evaluate the stress values in a multivariate manner and to look for diet related patterns. The PCA generate new variables that are a lineal combination of the previous ones, but in that case with the first PC explaining as much variance as possible.I It is a method for reducing information, very useful to look for pattern in the data.

## Results

**Fig 2:**
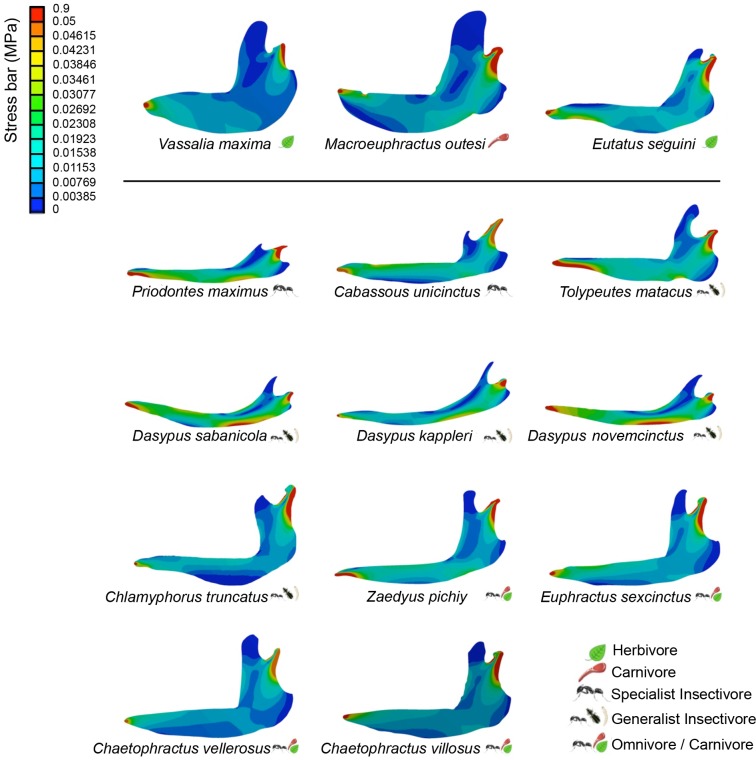
Patterns of Von Mises stress for the studied species under the boundary conditions of Set 1.

**Fig 3:**
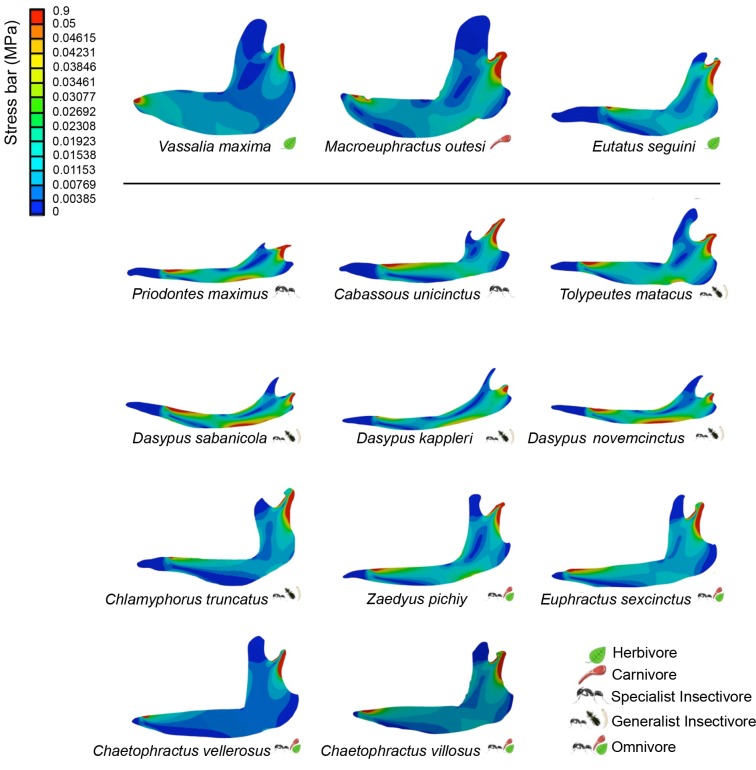
Patterns of Von Mises stress for the studied species under the boundary conditions of Set 2.

The visual representation of stress distribution for each mandible is a useful indicator for qualitative comparisons of their behavior under equivalent loads (Figs 2 and 3). Images from Von Mises models (Figs 2 and 3) show a pattern of colors that define areas according to the stress values: warm colors (red, orange and yellow) indicate region of high stress; bluish colors indicate little or no stress. Values recorded at each landmark are shown S2 and S3 Tables.

An unusually high stress appears where the boundary conditions are set as a simple support. These stresses are artificially inflated by the constraints imposed on the model due to a numerical singularity [54]. The numerical singularity is a consequence of the mathematical approach, and it is not related to any biological process. In those areas stresses have the tendency to increase the value towards infinity; therefore results of these areas are not considered.

### Von Mises stress distribution pattern

In Set 1 (Fig 2) all mandibles present a high level of stress at the mandibular notch, and from the condyle through the ramus in a descending direction.

#### Extant taxa

Specialist insectivore *Priodontes maximus* shows high stress at the ventral part of the tooth row. Both *P*. *maximus* and *C*. *unicinctus* have different biomechanical display along the mandible although the localization of some stressed areas coincides. The higher stress values are located at the anterior area of the mandible (without considering the inflated region due to the boundary conditions), which extends to the corpus being smaller at the posterior side of the mandible, taking very low values at the coronoid process.

Within the generalist insectivores (*Dasypus sabanicola*, *Dasypus kappleri*, *Dasypus novemcinctus*, *Tolypeutes matacus* and *Chlamyphorus truncatus*), all the members of the genus *Dasypus* displays similar patterns. However, it is not clear if this is a consequence of similarity in diet or phylogenetic signal, as all three species belong to the same subfamily. *D*. *novemcinctus* and *D*. *sabanicola* are the most alike with the highest values of stress at the anterior region of the mandible, which extends through the ventral part of the corpus until the mandible angle and goes up the ramus. Small values of stress are located at the upper area of the corpus mandible, where the upward shift leads to the ascending ramus. The sections with smallest stresses are the coronoid process and the elbow at the most posterior distal side of the mandible. *D*. *kappleri*, however, has little stress in the upper section of the corpus of the mandible and shows stress at the mandibular angle that extends towards the ramus shift as in the previously mentioned species, although less markedly. It also presents a slightly stressed region at the base of the proximal anterior side of the mandible, which extends through the corpus center. *T*. *matacus* shows a similar pattern.

In contrast, the specimen belonging to the genus *Chlamyphorus* shows a completely different pattern and, in general, smaller stress levels. In this taxon, areas with high stress are nonexistent except for the adjacent region of the condyle. This taxon also displays sections with a small stress from the base of the proximal anterior region of the mandible going along the corpus to the mandibular angle until the condyle. The regions with lower stresses are located at the basal area of the corpus and the coronoid process.

Regarding the omnivorous species (*Zaedyus pichiy*, *Euphractus sexcinctus*, *Chaetophractus vellerosus and Chaetophractus villosus*), these taxa have some coincident stress patterns. *Z*. *pichiy* and *E*. *sexcinctus* are the most similar considering the anterior part of the mandible; they show high levels of stress along the tooth row and slightly higher along the corpus with the basal region less stressed. *Z*. *pichiy* also presents values of stress at the mandibular angle, which is also present in specialist insectivores. In the case of the two *Chaetophractus* species, they display little stress levels compared to all other analyzed mandibles of extant species. This pattern is similar to those of the rest of the omnivores, which in general present less stress than insectivores.

#### Extinct taxa

*Vassallia maxima* shows almost no stress throughout the mandible. The posterior area of the mandible is the less affected, with exception of the high levels of stress in the adjacent zone of the condyle with the maximum incidence at the mandibular notch and descending towards the posterior distal part of the ramus.

*Macroeuphractus outesi* shows a peak of stress in the hollow situated in the anterior part of the mandible. However, this hollow is a broken part of the mandible. As it corresponds with the place where the caniniform should have rested it is not clear which was its original shape, thus we decided to not reconstruct it. Therefore, it is not possible to confirm if there is an actual peak of stress there, or if that is a consequence of a broken piece. Stress is also observed in the mandibular angle and the upper area of the corpus mandible, where the upward shift leads to the ascending ramus.

Among the fossils studied, *Eutatus seguini* shows the weakest mandible, and clearly presents higher stress values at the anterior basal region of the mandible that extends through the corpus with much less incidence to the coronoid process. The highest levels are situated at the mandibular notch and the area adjacent to the condyle. The sections with less stress are located at the anterior area of the mandible and to the upper part of the coronoid process.

For the Set 2 we obtained very similar results (Fig 3 and S3 Table).

### Statistical analysis

None of the Kruskal-Wallis tests (See S4 Table) was significant at 0.05, with exception of landmark 4 in Set 2. Therefore, no statistical differences between the different diets and stress levels were found.

**Fig 4:**
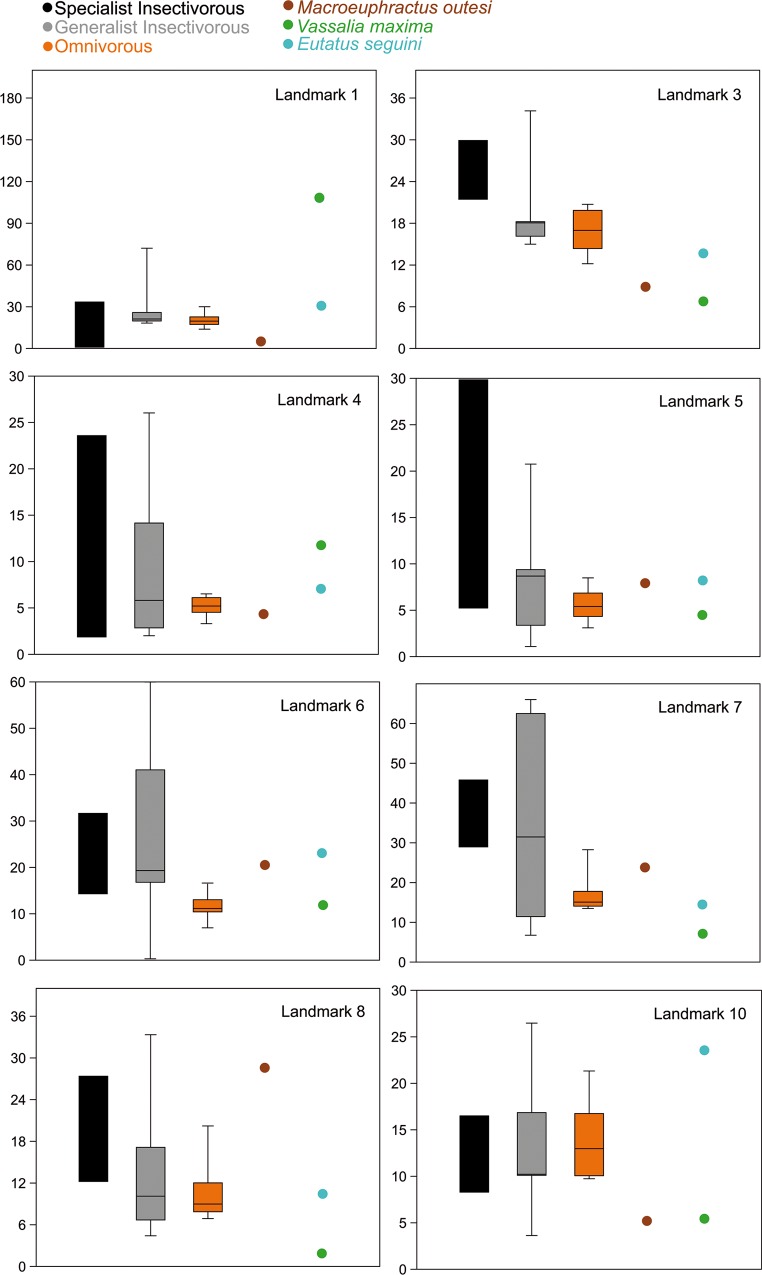
Box-plots of the Von Mises stress values for most of the landmarks for Set 1 (those with extreme outliers or affected by boundary conditions were excluded). Values of extant species are grouped by diet. The median is the middle line of the box and the whiskers represent the range of values.

However, despite the lack of significant results, some patterns appear in the box-plots for some landmarks (Fig 4). The most obvious result is that in general insectivores show a larger variance than omnivorous species. When median values are observed, omnivores have smaller stress values for most landmarks. Therefore, the absence of a significant result could be due to a lack of statistical power (large and heterogeneous variances plus small sample size).

Regarding extinct taxa, *Vassallia maxima* shows low values of stress along the mandible, with exception of the ventral part of the dentary. The most anterior part of the dentary should not be considered as it is affected by boundary conditions. *Eutatus*, the other species proposed as herbivorous, shows stress values within the range of insectivorous species.

In contrast, *Macroeuphractus outesi*, which has been described as a carnivorous species, presents stress values in the anterior part of the dentary smaller or within the range of omnivores. However, it displays higher values in the mandibular angle and the posterior end of the cheek row.

### Principal Component Analysis

Eigenvalues and loadings for each analysis can be found in S5 Table.

#### Set 1

Landmark 9 was omitted from the analysis, as the range of values was much higher than for the rest of the landmarks, hence drove all the variability. On the other hand, as this landmark was dependent on the situation of other landmarks, it seems not to reflect correctly an equivalent point between the different species. *Vassalia maxima* was excluded too from this analysis as its value for landmark 2 was a clear outlier, masking the distribution of the remaining species.

**Fig 5:**
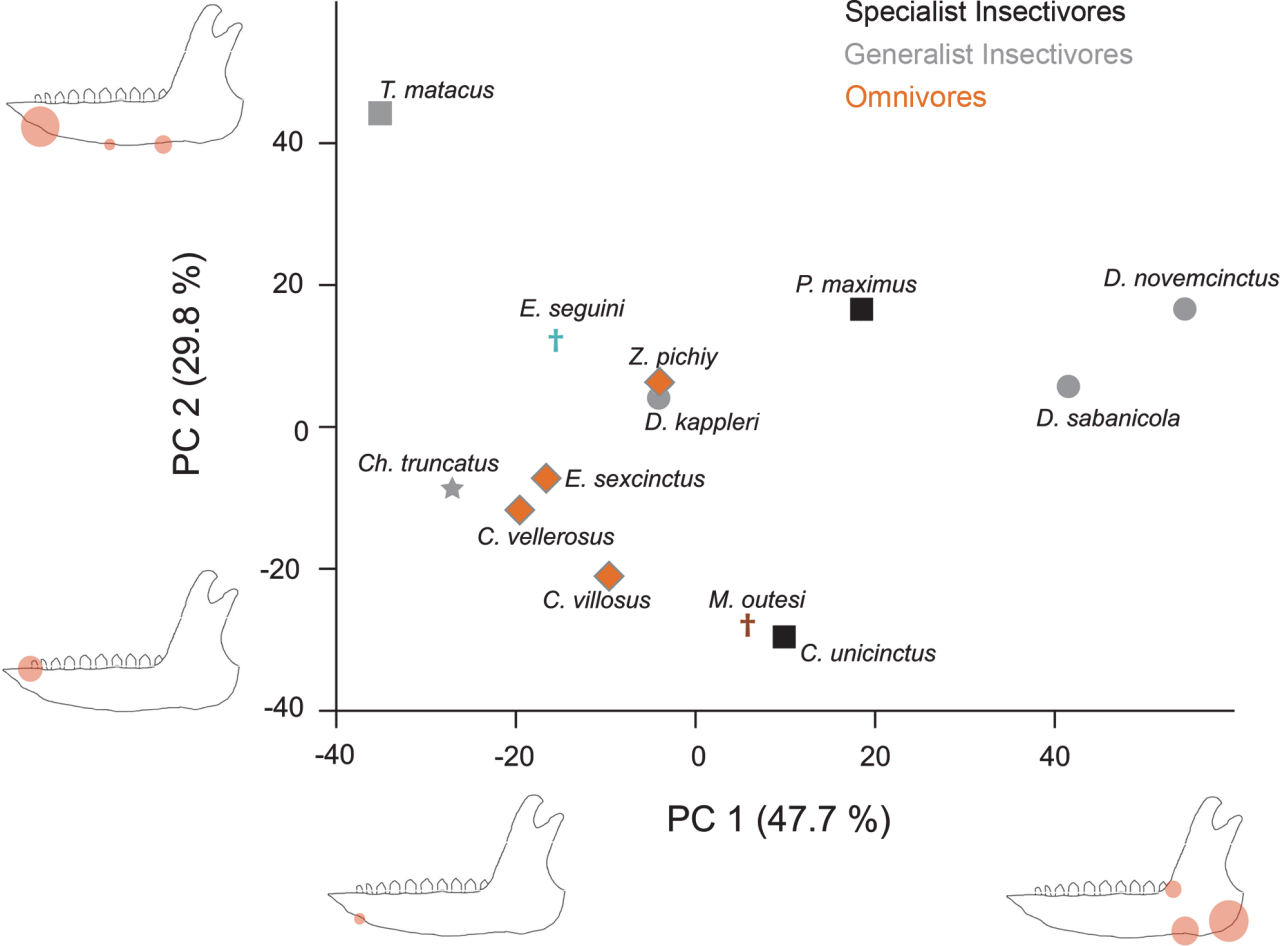
Dispersion graph of the two first principal components for Set 1. A hypothetical lower jaw is represented in each extreme of the axes, highlighting those landmarks with higher loadings in each PC (i.e., that have more importance in that PC) with red circles. The size of the circles is an approximation to the standardized value of the loading. Subfamilies are represented by different symbols; squares: Tolypeutinae, circles: Dasypodinae, diamonds: Euphractinae, stars: Chlamyphorinae.

The first principal component (hereafter, PC) explains almost 50% of the variance in the sample (S5 Table), and it is clearly dominated by *D*. *novemcinctus* and *D*. *sabanicola*, because of their high stress values at the mandibular angle (Fig 5). The negative part of the second PC (29.8% of the explained variance) has species with little stress in the most anterior and dorsal part of the jaw. The group of species in the positive part shows high stress values at the ventral border of the jaw, being higher in its anterior part (Fig 5). Following this, *T*. *matacus* shows the most stressed ventral area. Although there is no grouping by diet, the three omnivorous species, as well as *C*. *truncatus*, have negative values in both PCs, which in this case mean a global reduced stress although with the anterior part of the tooth row comparatively slightly more fragile.

#### Set 2

**Fig 6:**
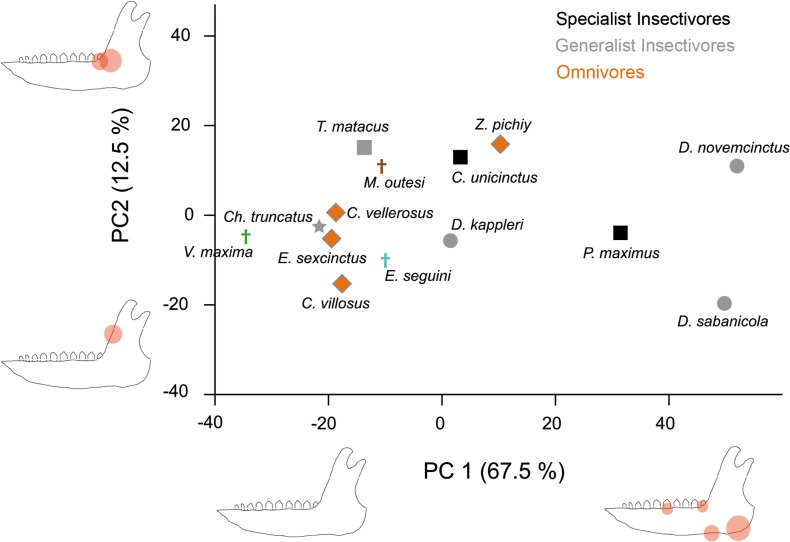
Dispersion graph of the two first principal components for Set 2. An hypothetical lower jaw is represented in each extreme of the axes, highlighting those landmarks with higher loadings in each PC (i.e., that have more importance in that PC) with red circles. The size of the circles is an approximation to the standardized value of the loading. Subfamilies are represented by different symbols; squares: Tolypeutinae, circles: Dasypodinae, diamonds: Euphractinae, stars: Chlamyphorinae.

The results for the first principal component are quite similar to those obtained in the Set 1, although the positive part shows intermediate stress values in the middle of the tooth row. The second PC, in opposition, explains a different pattern focused in the anterior part of the ascending ramus (Fig 6). Positive values are related to high stress values at the end of the tooth row and negative values imply high stress at the anterior part of the ascending ramus (Fig 6 and S5 Table). Again, there is no grouping by diet, not by phylogeny either, although the omnivorous and carnivorous species group together in the first PC, showing lower stress values with exception of *V*. *maxima*, which has the smaller ones following PC1.

## Discussion

### General Biomechanical Stress Patterns and diet

Our hypothesis was that high levels of stress along the mandible would represent a fragile mandible with a reduced capability for thoroughly chewing or processing hard items. On the other hand, low stress patterns would indicate a much more resistant mandible with a stronger capacity of oral processing. However, we have not found a significant correlation between diet and stress values, considering the sample analysed.

**Fig 7:**
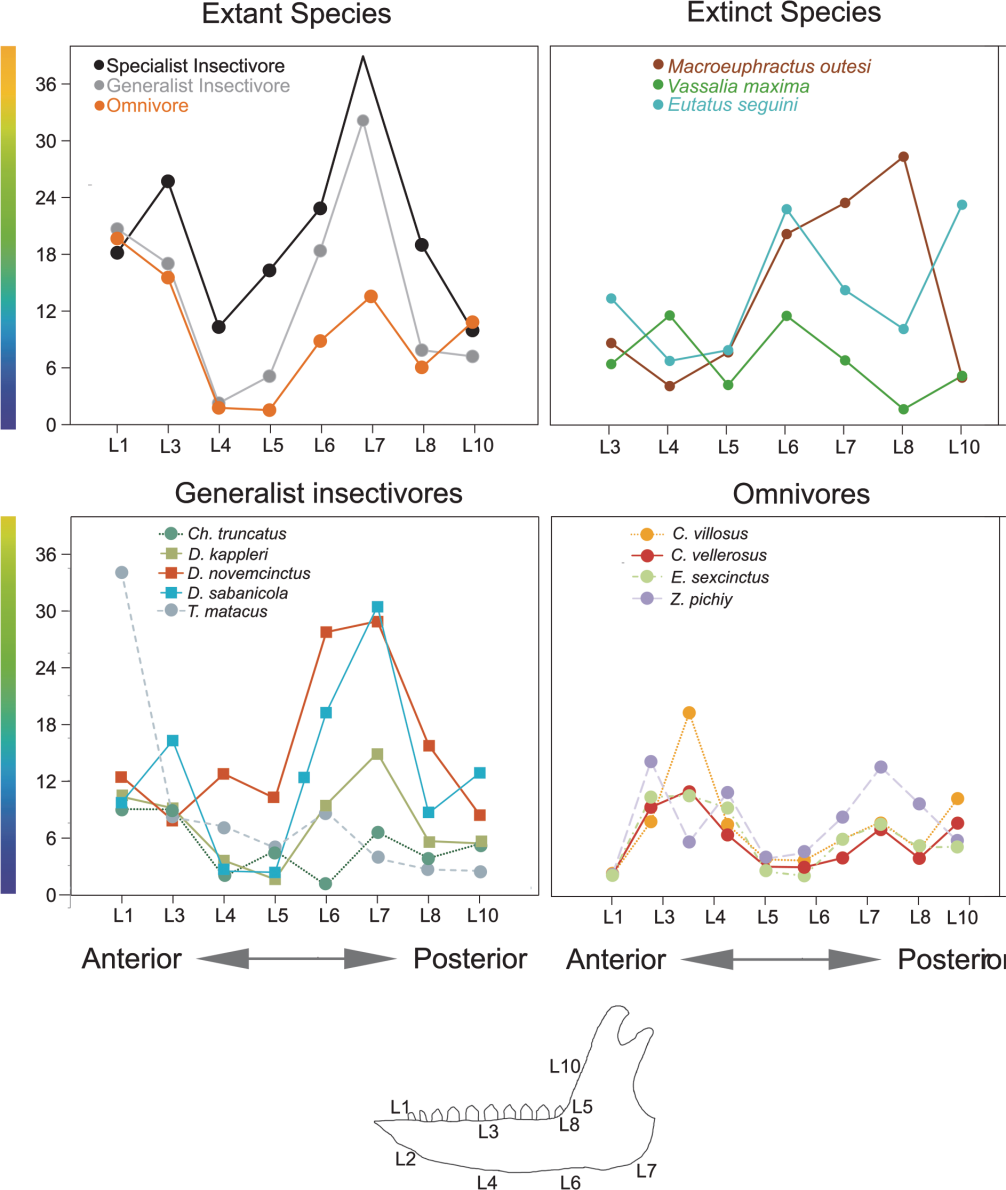
Line graph for the Von Mises stress values at each landmark, extant species grouped by diet.

Despite the lack of significant results, a general trend has appeared. As suggested in our biomechanical hypothesis, omnivores show smaller mean stress values for almost all landmarks (being landmark 10 an exception) and clearly reduced variability in their stress values (Figs 4 and 7), not showing peaks of great stress at any part of the jaw. *E*. *sexcinctus* is the only armadillo that can bite when handled [30] and it has been observed cracking palm nuts at the back of the mouth [15]. The FEA results for its mandible coincide with the expectations of a strong mandible. Moreover, some patterns have emerged in the analysis (Fig 7). Within extant species, the mandibular angle (Landmark 7) is one of the most fragile points (of the areas considered) in the mandible. Landmark 1 and 2 are too quite fragile, but it is hard to tell in those cases how much the boundary conditions, producing spurious stress levels, are affecting that zone.

As mentioned in the Results section, the lack of statistical significance could be due in part to the small sample size. However, this is not enough to explain the huge variability in the results of both groups of insectivorous species. Therefore, although some species show the biomechanical behavior expected for their proposed diet, others show stress patterns that do not agree with what was expected for the diet in which they had been classified.

### The exceptions: inaccurate FEA models or inaccurate proposed diets?

As mentioned above, some species display stress patterns that are not congruent with their traditional classification. This could be due to three different reasons; i) inaccurate FEA models, ii) incorrect diet classification, iii) a correct diet but processed in a different manner.

Both *Tolypeutes matacus* and *Chlamyphorus trunctatus* show stress at the recorded landmarks values well into the range of omnivorous species (Fig 7), with smaller values than the rest of insectivorous species. Some morphological characteristics of the lower jaw of *Tolypeutes matacus* have been described as traits shared with the omnivores [55]. These are a larger coronoid process and small posterior inclination of the ascending ramus. The stress pattern of this species is more similar to that of *Zaedyus pichiy*, which is reported to display a preference for the soft-bodied species such as larvae, tarantula spiders and pods [29]. *T*. *matacus* has been reported to have a very seasonal diet, with fruits and pods representing more than 50% of its diet during the dryer months [38]. Therefore, its diet might require some mouth processing after all, despite being an insectivorous species most of the year. On the other hand, it is worth mentioning that the landmarks recorded here do not capture the peak of high stress present at the ventral part of the mandibular angle in this species. This is a caveat against the interpretation of the FEA results based solely on a few points, the qualitative inspection of the colored maps (Figs 2 and 3) being important for the correct interpretation of the results.

*Chlamyphorus truncatus* is the other insectivorous species showing proportionally low values of stress at the recorded landmarks, and in general the stress pattern for the jaw looks more similar in colors (i.e. stress values) to omnivorous armadillos. This species have been reported to have a diet mainly composed of insects, although consuming worms, snails, and small amount of roots and other plants ([56], and references therein). However, it is not clear the frequency in which those items are consumed in the population. On the other hand, *C*. *truncatus* spends most of its life underground, tunneling through loose substrate in search for food [37]. Although it digs with its claws, this extreme life style could impose some constraints on the head shape, consequently affecting the shape of the jaw.

Finally, regarding insectivorous species, it is worth mentioning that *D*. *kappleri* have a jaw more resistant to forces than the two other species of the genera (*D*. *sabanicola* and *D*. *novemcinctus*) (Fig 7). Indeed, this species is grouped with the omnivorous taxa in the PCA (Figs 5 and 6). Although some vertebrate remains have been described in the stomach contents for this species [57], this is based in only one specimen. Moreover, vertebrate remains have been described in *D*. *novemcinctus* stomach contents, although accounting for a small fraction of its diet, and its mandible is the expected for an insectivorous species ([37], and references therein), thus exceptional intake of vertebrates would not explain a strong jaw. Otherwise, *D*. *kappleri* is the largest species of its family [35] and this might play a role on the biomechanical performance. Despite the scaling made here, allometric effects (change in shape as a consequence of change in size) have not been taken into account, which is a normal procedure in comparative FEA articles. As a consequence, more information on the diet of *D*. *kappleri*, as well as on the possible allometric effects observed here, will be needed to understand the reasons behind the smaller stress values for *D*. *kappleri* in comparison with the rest of *Dasypus* species.

*Zaedyus pichyi* shows the highest stress values among the omnivorous species (Fig 7), especially at the mandibular angle. Superina *et*. *al*. [58] report this species as “an opportunistic omnivore that mainly feed on insects and seems to be the least carnivorous of all carnivore-omnivore armadillos”. Thus the biomechanical results obtained here agree with the actual diet of the species.

The small number of extant species, as well as the lack of detailed knowledge of the diet of some of them, prevented other authors from developing a quantitative approach for the description of the diet in biomechanical and ecomorphological studies, creating instead broader categories. However, the approach followed here has been show to be sensitive enough to capture minor differences in diet for some species.

Possibility exists that the small variance of stress values recorded for each landmark in the omnivore/carnivore diet is related with the fact that all species showing this diet belong to the same subfamily. However, after detailed analysis of the diet of those insectivorous species contributing more to the high variance in this group are in the diet range of omnivores (see before). Whether this variance is a consequence of the phylogenetic variability of insectivores, dietary variability, or both, is something that should be addressed in the future including more species in the sample.

### Extinct taxa

The fossil taxon *Vassallia maxima* has been described primarily as a grazer consuming mainly coarse vegetation with morphological features shared with herbivorous ungulates [17–18]. In the same article those authors suggested that in this taxon the effective force bite is stronger at the most posterior tooth. Our biomechanical results agree with a strong mandible, as it shows a low stress pattern (Fig 6).

*Eutatus seguini* has been regarded as herbivorous based on the skull and dental morphology [9, 16]. Otherwise, the coronoid process and the position of the condyle are similar to those of the omnivorous *E*. *sexcintus*. Moreover, in *E*. *seguini* the coronoid process is inclined posteriorly as in insectivores and it has a remarkable large snout (characteristic shared with insectivores). The FEA results highlight combined characteristics for *E*. *seguini*, with a stress pattern similar to that in *M*. *outesi* in the anterior part and almost identical (although with higher values) to that in *V*. *maxima* in the posterior part (Fig 7). Therefore, even if corroborating the possibility of a behavior similar to that of *V*. *maxima* (although feeding on softer items as suggested by the higher stress values), the stress levels at the anterior part of the jaw indicate that probably it fed too in other kind of items.

The extinct euphractine *Macroeuphractus outesi* has been biomechanically analyzed in previous works [28]. Those authors concluded that this species has a powerful and fast anterior bite and a powerful backside bite condition. The pattern and levels of stress we have found in this study are different from those extant omnivorous species analyzed here (Fig 6) and also from any other diet studied in this work, although indeed more similar to insectivorous species in the posterior part of the jaw. Therefore, although it might have been a carnivorous species, more work is needed in a comparative framework including its diet to corroborate it.

Despite more research has to be done in relation to quantitative analysis of FEA data and the allometric and phylogenetic effects on the results, the differences between the stress patterns that were not congruent with the diet category could be explained under a detailed analysis of the diet of each species. This finding supports the use of FEA models as suitable predictors of biomechanical behavior in ecomorphological studies in armadillos, at least to differentiate between insectivorous and omnivorous species. For detailed feeding predictions more work is needed accounting for the effect of factors other than diet on the lower jaw (e.g. life style, allometry).

## Conclusions

The results of finite elements analysis (FEA) of the mandibles of several genera of Cingulata have shown to be related, in general terms, to the diet of these species. The difference between omnivorous and insectivorous species is clear. Those species that do not completely met the expectations of stress level for their diet can be explained with a more detailed study of their diet. *D*. *kappleri* has a lower jaw more resistant than its genera counterparts. However it remains unclear whether this is due to a different diet, an allometric effect, or other factors.

For the extinct species studied, all of them outside the range of insectivorous diet, some conclusions can be derived. The fossil taxon *Vassallia maxima* presented a very strong mandible, congruent with an herbivorous diet with a great amount of oral processing and in agreement with Vizcaíno *et al*. [17] morphofunctional studies. On the other hand, *Eutatus seguini* shows a stress pattern similar to *Vassalia* in the posterior part, but to *Macroeuphractus outesi* in the anterior part of its very strong mandible. This last species display a strong mandible.

FEA analysis on planar models has shown as a powerful tool for biomechanical studies in a comparative framework within Cingulata.

## Supporting Information

S1 Table Von-Mises stress values at each landmark for set 1. The values measured in each landmark are multiplied for a thousand.
(DOC)

S2 Table Von-Mises stress values at each landmark for set 2. The values measured in each landmark are multiplied for a thousand.
(DOC)

S3 Table P-values for the Kruskall-Wallis analysis for each landmark and set. (DOC)

S4 Table Eigenvalues of the PCAs developed with the values of stress at each landmark and set. (DOC)

S5 Table Loadings of the PCAs developed with the values of stress at each landmark and set. (DOC)
