# Correction: Finite Element Analysis of the Cingulata Jaw: An Ecomorphological Approach to Armadillo's Diets

**DOI:** 10.1371/journal.pone.0129953

**Published:** 2015-06-03

**Authors:** The PLOS ONE Staff

The Academic Editor field does not appear on this article. The Academic Editor is Stuart Humphries, University of Lincoln, United Kingdom. The publisher apologizes for the error.
