# Supplementary material for: Finite Element Analysis of the Cingulata Jaw: An Ecomorphological Approach to Armadillo’s Diets
Source: PLoS One. 2015 Apr 28;10(4):e0120653. doi: 10.1371/journal.pone.0120653 (PMC4412537; doi:10.1371/journal.pone.0120653)
Supplement: S1 Table — The values measured in each landmark are multiplied for a thousand. (DOC) [file pone.0120653.s001.doc]

| Species | Von-Mises Stress (MPa) | | | | | | | | | |
| --- | --- | --- | --- | --- | --- | --- | --- | --- | --- | --- |
| Set 1 | Landmark 1 | Landmark 2 | Landmark 3 | Landmark 4 | Landmark 5 | Landmark 6 | Landmark 7 | Landmark 8 | Landmark 9 | Landmark 10 |
| *Chaetophractus villosus* | 15.69 | 41.86 | 15.02 | 6.61 | 6.36 | 11.47 | 15.66 | 8.98 | 269.94 | 21.24 |
| *Priodontes maximus* | 35.19 | 25.56 | 21.50 | 23.58 | 5.31 | 31.65 | 45.80 | 12.28 | 16.02 | 16.50 |
| *Cabassous unicinctus* | 3.10 | 35.12 | 29.14 | 1.88 | 29.93 | 14.31 | 29.35 | 27.37 | 635.14 | 8.38 |
| *Chlamyphorus truncatus* | 18.27 | 8.48 | 18.21 | 2.33 | 8.48 | 0.29 | 12.86 | 6.71 | 8.41 | 10.09 |
| *Chaetophractus vellerosus* | 19.15 | 23.17 | 12.51 | 4.88 | 4.74 | 6.98 | 14.07 | 6.89 | 9.67 | 15.34 |
| *Dasypus kapplery* | 21.20 | 6.18 | 18.49 | 6.15 | 1.74 | 19.32 | 31.47 | 10.73 | 13.77 | 10.19 |
| *Dasypus novemcinctus* | 25.94 | 26.94 | 15.65 | 26.56 | 20.80 | 59.96 | 62.53 | 33.34 | 13.97 | 16.85 |
| *Dasypus sabanicola* | 19.79 | 24.69 | 34.41 | 3.67 | 3.38 | 41.03 | 66.04 | 17.32 | 13.04 | 26.81 |
| *Euphractus sexcinctus* | 21.60 | 21.98 | 18.98 | 3.72 | 2.69 | 11.57 | 15.09 | 9.51 | 10.74 | 9.66 |
| *Tolypeutes matacus* | 74.28 | 5.93 | 16.32 | 14.20 | 9.15 | 17.64 | 6.62 | 4.03 | 752.40 | 3.72 |
| *Zaedyus pichiy* | 30.22 | 10.82 | 22.70 | 6.79 | 8.19 | 16.79 | 28.82 | 19.98 | 10.96 | 10.96 |
| *Vassallia maxima* | 107.93 | 55.96 | 6.67 | 11.82 | 4.46 | 11.77 | 7.07 | 1.90 | 9.47 | 5.43 |
| *Eutatus seguini* | 30.15 | 0.26 | 13.61 | 7.00 | 8.15 | 23.05 | 14.49 | 10.38 | 13.40 | 23.51 |
| *Macroeuphractus outesi* | 4.934 | 41.86 | 8.89 | 4.35 | 7.92 | 20.42 | 23.71 | 28.59 | 14.86 | 5.25 |
