# Supplementary material for: Finite Element Analysis of the Cingulata Jaw: An Ecomorphological Approach to Armadillo’s Diets
Source: PLoS One. 2015 Apr 28;10(4):e0120653. doi: 10.1371/journal.pone.0120653 (PMC4412537; doi:10.1371/journal.pone.0120653)
Supplement: S2 Table — The values measured in each landmark are multiplied for a thousand. (DOC) [file pone.0120653.s002.doc]

| Species | Von-Mises Stress (MPa) | | | | | | | | |
| --- | --- | --- | --- | --- | --- | --- | --- | --- | --- |
| Set 2 | Landmark 1 | Landmark 3 | Landmark 4 | Landmark 5 | Landmark 6 | Landmark 7 | Landmark 8 | Landmark 9 | Landmark 10 |
| *Chaetophractus villosus* | 26.56 | 16.83 | 3.92 | 6.21 | 13.32 | 17.03 | 3.96 | 261.65 | 22.01 |
| *Priodontes maximus* | 24.00 | 31.89 | 6.10 | 7.91 | 35.16 | 51.95 | 31.10 | 14.31 | 24.00 |
| *Cabassous unicinctus* | 20.30 | 39.76 | 9.84 | 28.93 | 11.10 | 29.48 | 25.51 | 593.56 | 8.62 |
| *Chlamyphorus truncatus* | 12.01 | 22.05 | 5.28 | 3.33 | 3.46 | 18.41 | 15.99 | 8.82 | 10.60 |
| *Chaetophractus vellerosus* | 20.39 | 14.65 | 1.41 | 12.93 | 8.81 | 16.01 | 16.69 | 9.99 | 15.50 |
| *Dasypus kapplery* | 14.13 | 27.49 | 7.47 | 2.73 | 18.53 | 34.46 | 20.45 | 12.12 | 15.55 |
| *Dasypus novemcinctus* | 32.42 | 28.67 | 7.21 | 23.72 | 55.75 | 62.77 | 40.49 | 11.74 | 19.36 |
| *Dasypus sabanicola* | 37.80 | 47.13 | 12.03 | 6.66 | 37.30 | 67.00 | 24.29 | 10.53 | 32.12 |
| *Euphractus sexcinctus* | 14.79 | 22.54 | 2.38 | 11.50 | 14.32 | 17.58 | 4.10 | 11.26 | 9.79 |
| *Tolypeutes matacus* | 28.71 | 18.53 | 7.58 | 11.21 | 22.82 | 7.63 | 29.73 | 604.71 | 4.05 |
| *Zaedyus pichiy* | 18.93 | 27.84 | 1.92 | 22.52 | 23.71 | 35.65 | 32.17 | 32.17 | 7.18 |
| *Vassallia maxima* | 14.86 | 6.80 | 11.56 | 4.06 | 12.18 | 7.28 | 2.59 | 9.48 | 5.35 |
| *Eutatus seguini* | 14.69 | 15.75 | 2.05 | 5.76 | 28.31 | 16.99 | 13.18 | 13.89 | 26.13 |
| *Macroeuphractus outesi* | 11.39 | 10.62 | 2.85 | 8.21 | 19.81 | 23.40 | 28.82 | 14.52 | 5.18 |
