# Supplementary material for: Finite Element Analysis of the Cingulata Jaw: An Ecomorphological Approach to Armadillo’s Diets
Source: PLoS One. 2015 Apr 28;10(4):e0120653. doi: 10.1371/journal.pone.0120653 (PMC4412537; doi:10.1371/journal.pone.0120653)
Supplement: S3 Table — (DOC) [file pone.0120653.s003.doc]

| Set 1 | Landmark 1 | Landmark 2 | Landmark 3 | Landmark 4 | Landmark 5 | Landmark 6 | Landmark 7 | Landmark 8 |  | Landmark 10 |
| --- | --- | --- | --- | --- | --- | --- | --- | --- | --- | --- |
| H (chi^2^): | 1.2610 | 0.9697 | 2.7150 | 0.2424 | 2.5940 | 3.3820 | 2.0120 | 1.7450 |  | 2.0610 |
| p-value: | 0.5324 | 0.6158 | 0.2573 | 0.8858 | 0.2734 | 0.1844 | 0.3657 | 0.4178 |  | 0.3569 |
| Set 2 | Landmark 1 | Landmark 2 | Landmark 3 | Landmark 4 | Landmark 5 | Landmark 6 | Landmark 7 | Landmark 8 | Landmark 9 | Landmark 10 |
| H (chi^2^): | 0.3758 | - | 4.1700 | 4.6790 | 0.7758 | 2.1700 | 4.7760 | 5.2120 | 4.1940 | 0.6788 |
| p-value: | 0.8287 | - | 0.1243 | 0.0964 | 0.6785 | 0.3380 | 0.0918 | 0.0738 | 0.1228 | 0.7122 |
