# Supplementary material for: Finite Element Analysis of the Cingulata Jaw: An Ecomorphological Approach to Armadillo’s Diets
Source: PLoS One. 2015 Apr 28;10(4):e0120653. doi: 10.1371/journal.pone.0120653 (PMC4412537; doi:10.1371/journal.pone.0120653)
Supplement: S4 Table — (DOC) [file pone.0120653.s004.doc]

| SET1 | | | SET2 | | |
| --- | --- | --- | --- | --- | --- |
| PC | Eigenvalue | % variance | PC | Eigenvalue | % variance |
| 1 | 675.682 | 47.716 | 1 | 709.752 | 67.615 |
| 2 | 421.685 | 29.779 | 2 | 131.811 | 12.557 |
| 3 | 128.246 | 9.0566 | 3 | 97.0947 | 9.2497 |
| 4 | 80.2454 | 5.6668 | 4 | 46.7411 | 4.4528 |
| 5 | 56.3504 | 3.9794 | 5 | 27.6292 | 2.6321 |
| 6 | 30.6522 | 2.1646 | 6 | 20.3614 | 1.9397 |
| 7 | 17.4652 | 1.2334 | 7 | 12.0106 | 1.1442 |
| 8 | 3.8014 | 0.26845 | 8 | 4.30182 | 0.40981 |
