# Supplementary material for: Finite Element Analysis of the Cingulata Jaw: An Ecomorphological Approach to Armadillo’s Diets
Source: PLoS One. 2015 Apr 28;10(4):e0120653. doi: 10.1371/journal.pone.0120653 (PMC4412537; doi:10.1371/journal.pone.0120653)
Supplement: S5 Table — (DOC) [file pone.0120653.s005.doc]

| SET1 | PC 1 | PC 2 | PC 3 | PC 4 | PC 5 | PC 6 | PC 7 | PC 8 | PC 9 |
| --- | --- | --- | --- | --- | --- | --- | --- | --- | --- |
| Landmark 1 | -0.205 | 0.780 | 0.323 | 0.131 | 0.338 | 0.000 | -0.215 | 0.171 | -0.195 |
| Landmark 2 | 0.230 | -0.436 | 0.587 | 0.551 | 0.310 | 0.027 | -0.099 | -0.037 | -0.030 |
| Landmark 3 | 0.110 | 0.011 | -0.284 | -0.103 | 0.688 | 0.129 | -0.050 | -0.001 | 0.635 |
| Landmark 4 | 0.142 | 0.240 | 0.305 | 0.090 | -0.203 | 0.023 | 0.753 | 0.232 | 0.397 |
| Landmark 5 | 0.100 | -0.074 | 0.311 | -0.579 | 0.227 | 0.553 | 0.223 | -0.217 | -0.312 |
| Landmark 6 | 0.527 | 0.316 | 0.141 | -0.006 | -0.360 | 0.138 | -0.366 | -0.481 | 0.298 |
| Landmark 7 | 0.705 | 0.142 | -0.320 | 0.061 | 0.235 | -0.271 | 0.225 | 0.073 | -0.439 |
| Landmark 8 | 0.275 | -0.133 | 0.267 | -0.450 | -0.104 | -0.171 | -0.357 | 0.671 | 0.121 |
| Landmark 10 | 0.116 | 0.023 | -0.295 | 0.345 | -0.165 | 0.744 | -0.109 | 0.425 | -0.077 |
| SET2 |  |  |  |  |  |  |  |  |  |
|  | PC 1 | PC 2 | PC 3 | PC 4 | PC 5 | PC 6 | PC 7 | PC 8 |  |
| Landmark 1 | 0.214 | -0.090 | -0.043 | 0.709 | 0.460 | -0.071 | -0.430 | -0.202 |  |
| Landmark 3 | 0.323 | -0.088 | 0.663 | 0.129 | 0.144 | 0.071 | 0.586 | -0.250 |  |
| Landmark 4 | 0.044 | -0.046 | 0.088 | 0.096 | 0.271 | -0.498 | 0.180 | 0.791 |  |
| Landmark 5 | 0.112 | 0.490 | 0.254 | 0.452 | -0.627 | 0.097 | -0.130 | 0.245 |  |
| Landmark 6 | 0.445 | 0.056 | -0.665 | 0.193 | -0.186 | -0.178 | 0.486 | -0.130 |  |
| Landmark 7 | 0.707 | -0.176 | 0.153 | -0.395 | -0.191 | -0.257 | -0.432 | -0.017 |  |
| Landmark 8 | 0.317 | 0.634 | -0.094 | -0.246 | 0.471 | 0.422 | -0.015 | 0.168 |  |
| Landmark 10 | 0.193 | -0.553 | -0.104 | 0.120 | -0.094 | 0.675 | 0.017 | 0.408 |  |
